# Trichoscopy findings in dissecting cellulitis^☆^^☆☆^

**DOI:** 10.1016/j.abd.2019.09.006

**Published:** 2019-09-30

**Authors:** Daniel Fernandes Melo, Erica Bertolace Slaibi, Thais Marques Feitosa Mendes Siqueira, Violeta Duarte Tortelly

**Affiliations:** Alopecia Outpatient Clinic, Hospital Naval Marcílio Dias, Rio de Janeiro, RJ, Brazil

**Keywords:** Alopecia, Cellulitis, Dermoscopy, Folliculitis, Hair, Scalp dermatoses

## Abstract

Dissecting cellulitis is an inflammatory, chronic, and recurrent disease of the hair follicles that mainly affects young Afro-descendent men. Trichoscopy is a method of great diagnostic value for disorders of the scalp. Clinical and trichoscopic findings of dissecting cellulitis are heterogeneous and may present features common to non-cicatricial and scarring alopecia. This article presents the trichoscopic findings of dissecting cellulitis that help in the diagnosis and consequent institution of the appropriate therapy and better prognosis of the disease.

## Introduction

Dissecting cellulitis (DC), also called folliculitis abscedens or perifolliculitis capitis abscedens et suffodiens, is an inflammatory, chronic, and recurrent disease of the hair follicles, with uncertain etiopathogenesis and probable genetic influence, which can be triggered by environmental factors.^1^ It predominantly affects young Afro-descendent men, at the vertex and occipital region.^1^, ^2^

Initially, papulopustular lesions evolve with the formation of areas of non-cicatricial alopecia and later, multifocal painful nodules and interconnected abscesses, which may or may not fistulize. If the inflammatory process is not contained or there are frequent recurrences, there will be areas of scarring alopecia with aesthetic and psychosocial impairment for the patient.^3^, ^4^

Trichoscopy is a practical, useful, and non-invasive method that has shown great value in a range of disorders of the scalp and hair shaft.^3^, ^4^ Trichoscopic findings contribute to early diagnosis, execution of guided biopsy, and consequent appropriate choice of therapy and follow-up of cases with potential evolution to cicatricial alopecia.

Given the high prevalence of Afro-descendants in Brazil, the increasing recognition of cases, and the scarcity of publications on the subject, the purpose of this article is to enumerate and detail, in a didactic way, the trichoscopic findings of DC. The aim is to contribute to the diagnosis and, eventually, to modify the disfiguring scarring course that is characteristic of the disease.

## Discussion

The heterogeneity of the clinical and trichoscopic findings of DC is explained by the recurrence of the inflammatory process in the same patient over the same area.^4^, ^5^

In early stages of the disease, the inflammatory component is less exuberant and trichoscopy may, on this occasion, resemble that of patchy non-cicatricial alopecia, and alopecia areata represents an important differential diagnosis.^3^, ^4^ In this stage, broken hair shafts of variable length can be found, as well as black dots, corresponding to lumps of keratin resulting from the breaking of shafts at the emergence of the follicular ostium.^5^ Although controversial, the presence of exclamation mark hairs^4^ and circle hairs in the initial stages of DC has also been described.^6^, ^7^

Yellow dots represent sebum accumulation and keratin in the follicular infundibulum and, usually when found in DC, are large in size, yellowish-brown in color, double-bordered, and may or may not contain dystrophic shafts. These characteristics confer the typical three-dimensional (“3D”) or “soap bubble” aspect to this yellow dot, which represents the most specific trichoscopic finding of DC.^8^, ^9^, ^10^ Empty follicular openings, better evaluated by dermoscopy, seem to be related with a better prognosis for hair regrowth, since they are viable hair follicles. At this point, institution of adequate treatment confers the possibility of non-progression to an irreversible cicatricial stage.^4^, ^5^, ^8^

In the presence of a more exuberant inflammatory process, in an abscessing phase *per se*, peri- and interfollicular scales and erythema in varying degrees can be seen at trichoscopy. A disrupted yellow area and pustules can be seen on DC and represent true pus lakes surrounding the follicular openings, which later give rise to infection and even hematic crusts if there is associated local trauma.^10^ Large dark brown follicular openings (large brown dots), with the appearance of comedones, were also observed by Abedini et al.^10^ Such structures are commonly seen and are characteristic of DC, corroborating the fact that this condition is inserted in the context of diseases caused by follicular obstruction, such as acne conglobata, hidradenitis suppurativa, and pilonidal cyst.^1^, ^2^

Polytrichia, which represents the emergence of five or more shafts per follicular unit, may be present in later stages of the disease.^5^, ^6^, ^8^ The same occurs with skin clefts with emergent hairs, corresponding to skin folds containing shafts.^10^ Empty follicular units replaced by fibrosis represented by white dots and amorphous white areas can also be visualized in advanced forms of the disease, where the fibrotic component prevails.^5^, ^6^, ^8^ Additional findings already described include blue-gray dots with histopathological correspondence to pigmentary incontinence and nonspecific vascular signs, such as punctate vessels and red dots.^6^, ^7^, ^10^ The presence of short regrowing hairs is indicative of periods of remission, often found in early phases of the disease, while the scarring areas denote late stages with a recurrent poor response to clinical therapy.^4^, ^5^

**Figure 1:**
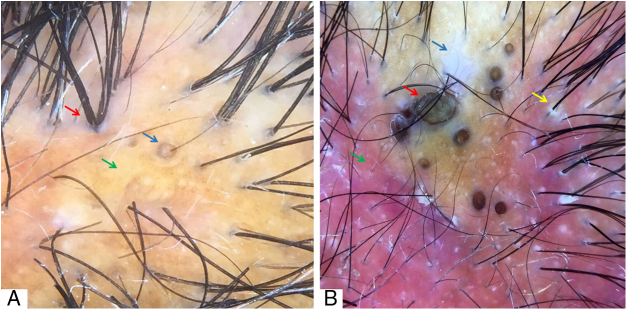
(A) “3D” yellow dot (blue arrow), polytrichia (red arrow), and yellow areas (green arrow). (B) Amorphous white area (blue arrow), large brown dots (red arrow), diffuse erythema (green arrow), perifollicular scales (yellow arrow). Trichoscopy performed with 3Gen DermLite® II Hybrid M with polarized light and interface liquid (A) and without interface liquid (B) (alcohol 70%); ×20 magnification.

**Figure 2:**
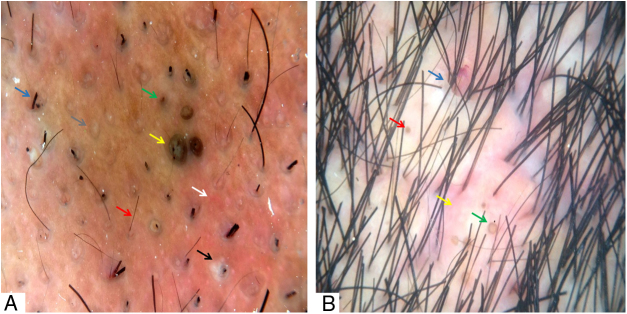
(A) Broken hairs (blue arrow), short regrowing hairs (red arrow), black dots (green arrow), large brown dots (yellow arrow), follicular pustules (black arrow), interfollicular erythema (white arrow), and empty follicular openings (gray arrow). (B) Skin clefts with emergent hairs (blue arrow), yellow dots (red arrow), “3D” yellow dots (green arrow), and peri- and interfollicular erythema (yellow arrow). Trichoscopy performed with 3Gen DermLite® II Hybrid M with polarized light with interface liquid (alcohol 70%); ×20 magnification.

**Figure 3:**
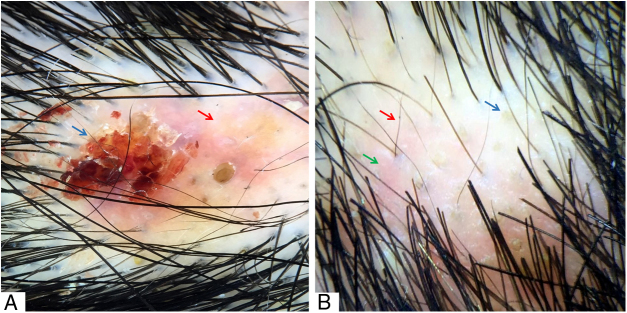
(A) Hematic crust (blue arrow) and erythematous-yellowish area (red arrow). (B) Yellow dots (blue arrow), short regrowing hairs (red arrow), and interfollicular erythema (green arrow). Trichoscopy performed with 3Gen DermLite® II Hybrid M with polarized light and with interface liquid (alcohol 70%); ×20 magnification.

**Figure 4:**
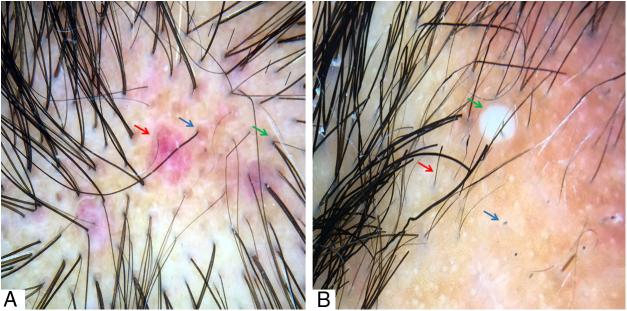
(A) Red dots (blue arrow), peri- and interfollicular erythema (red arrow), and perifollicular gray-blue pigmentation (green arrow). (B) Black dots (blue arrow), exclamation mark, and dystrophic hairs (red arrow) and pustule (green arrow) Trichoscopy performed with 3Gen DermLite® II Hybrid M with polarized light and with interface liquid (70% alcohol); ×20 magnification.

The major trichoscopic findings of DC and their representative images are shown in Table 1 and Figure 1, Figure 2, Figure 3, Figure 4, respectively.

**Table 1:** Trichoscopic findings of dissecting cellulitis.

01	Broken hair	11	Yellowish, hematic crusts
02	Black dots	12	Large brown dots
03	Exclamation mark hairs	13	Polytrichia
04	Circular hairs	14	Cutaneus clefts with emerging hairs
05	Yellow dots	15	White dots
06	3D yellow dots (soap bubble)	16	Amorphous white areas
07	Empty follicular openings	17	Blue-gray dots
08	Peri- and interfollicular scales	18	Punctate vessels
09	Erythema	19	Red dots
10	Pustules and structureless yellow areas	20	Short regrowing hairs

## Final considerations

There are many trichoscopic findings of DC and they may be heterogeneous and even overlapped throughout the evolution of the disease. Although not pathognomonic, recognition of the trichoscopic alterations already described is important and more studies are needed to determine the sensitivity and specificity of these findings in this clinical condition. Therefore, the use of trichoscopy, a non-invasive technique with rapid application, when associated with good clinical evaluation, increases the diagnostic accuracy and allows a better follow-up and prognosis for the affected patients.

## Author's contribution

Daniel Fernandes Melo: Approval of the final version of the manuscript; conception and planning of the study; elaboration and writing of the manuscript; obtaining, analyzing and interpreting the data; effective participation in research orientation; intellectual participation in propaedeutic and/or therapeutic conduct of the cases studied; critical review of the literature; critical review of the manuscript.

Erica Bertolace Slaibi: Elaboration and writing of the manuscript; critical review of the literature; critical review of the manuscript.

Thais Marques Feitosa Mendes Siqueira: Elaboration and writing of the manuscript; critical review of the literature; critical review of the manuscript.

Violeta Duarte Tortelly: Approval of the final version of the manuscript; conception and planning of the study; elaboration and writing of the manuscript; obtaining, analyzing and interpreting the data; effective participation in research orientation; intellectual participation in propaedeutic and/or therapeutic conduct of the cases studied; critical review of the literature; critical review of the manuscript.

## Financial support

None.

## Conflicts of interest

The authors declare no conflicts of interest.
